# Insights into Hepatopancreatic Functions for Nutrition Metabolism and Ovarian Development in the Crab *Portunus trituberculatus*: Gene Discovery in the Comparative Transcriptome of Different Hepatopancreas Stages

**DOI:** 10.1371/journal.pone.0084921

**Published:** 2014-01-13

**Authors:** Wei Wang, Xugan Wu, Zhijun Liu, Huajun Zheng, Yongxu Cheng

**Affiliations:** 1 Key Laboratory of Exploration and Utilization of Aquatic Genetic Resources, Shanghai Ocean University, Ministry of Education, Shanghai, China; 2 Chinese National Human Genome Center at Shanghai, Shanghai, China; International Centre for Genetic Engineering and Biotechnology, Italy

## Abstract

The crustacean hepatopancreas has different functions including absorption, storage of nutrients and vitellogenesis during growth, and ovarian development. However, genetic information on the biological functions of the crustacean hepatopancreas during such processes is limited. The swimming crab, *Portunus trituberculatus*, is a commercially important species for both aquaculture and fisheries in the Asia-Pacific region. This study compared the transcriptome in the hepatopancreas of female *P. trituberculatus* during the growth and ovarian maturation stages by 454 high-throughput pyrosequencing and bioinformatics. The goal was to discover genes in the hepatopancreas involved in food digestion, nutrition metabolism and ovarian development, and to identify patterns of gene expression during growth and ovarian maturation. Our transcriptome produced 303,450 reads with an average length of 351 bp, and the high quality reads were assembled into 21,635 contigs and 31,844 singlets. Based on BLASTP searches of the deduced protein sequences, there were 7,762 contigs and 4,098 singlets with functional annotation. Further analysis revealed 33,427 unigenes with ORFs, including 17,388 contigs and 16,039 singlets in the hepatopancreas, while only 7,954 unigenes (5,691 contigs and 2,263 singlets) with the predicted protein sequences were annotated with biological functions. The deduced protein sequences were assigned to 3,734 GO terms, 25 COG categories and 294 specific pathways. Furthermore, there were 14, 534, and 22 identified unigenes involved in food digestion, nutrition metabolism and ovarian development, respectively. 212 differentially expressed genes (DEGs) were found between the growth and endogenous stage of the hepatopancreas, while there were 382 DEGs between the endogenous and exogenous stage hepatopancreas. Our results not only enhance the understanding of crustacean hepatopancreatic functions during growth and ovarian development, but also represent a basis for further research on new genes and functional genomics of *P. trituberculatus* or closely related species.

## Introduction

In crustaceans, the hepatopancreas is an important organ for the absorption and storage of nutrients, and can synthesize digestive enzymes for food digestion [1], [2]. The stored nutrients are transported to the muscle, gonads and other tissues during the growth and reproductive stages [3], [4]. Some crustaceans can store large amounts of energy, particularly lipids, in the hepatopancreas for energy expenditure during molting, starvation, or reproduction [5], [6]. Furthermore, the hepatopancreas is an important site for the synthesis of vitellogenin and sex steroid hormones, and for some biosynthetic steps in these pathways [7]–[9]. Therefore, the crustacean hepatopancreas plays important roles during growth and ovarian maturation [2], [10]. However, there are no comparisons at a molecular level of hepatopancreatic function during the growth and ovarian development stages of crustaceans.

The swimming crab *Portunus trituberculatus* is a commercially important species widely distributed in the coastal waters of East Asian countries, particularly in China, Korea and Japan [11]. Because of the high nutritional value and increasing market demands for this species, pond-culture of *P. trituberculatus* has developed and spread quickly in the east coast regions of China since the 1990s [12]. The life cycle of *P. trituberculatus* is around 2–3 years, and they can reach sexual maturity during the first year [13]. Before the puberty molt, *P. trituberculatus* grows quickly via frequent molting (every 5–30 days). During the growth stage, the main function of the hepatopancreas is to digest, assimilate, and store nutrients for tissue growth and energy consumption [3], [14]. After the puberty molt and mating, female *P. trituberculatus* usually start vitellogenesis and ovarian development, which can be divided into two stages, the endogenous vitellogenic stage, and exogenous vitellogenic stage [15]. Our previous study has shown that the hepatopancreas of *P. trituberculatus* is a major site for exogenous vitellogenesis [16]. Clearly, the hepatopancreas is involved in growth and ovarian development of *P. trituberculatus*, and makes a significant contribution to these processes. However, a detailed understanding of the molecular mechanisms of these processes is unknown.

To date, genomic sequence resources available for *P. trituberculatus* are quite limited. Only thousands of nucleotide sequences are available in the public databases, including 14,340 expressed sequence tags (ESTs) from three cDNA libraries of gill, eyestalk and hemolymph from *P. trituberculatus* [17], [18]. Because of the redundancy rate of crab EST, the unique genes represented by these ESTs are relatively small. Therefore, the lack of genetic information has restricted our understanding of the roles of the hepatopancreas of *P. trituberculatus* in nutrition metabolism, ovarian development, and other physiological activities. However, next generation sequencing technology, such as Roche 454 and Illumina Solexa platforms, has revolutionized biological research by providing genomic and transcriptomic data cheaply and rapidly for expanding the sequence databases of non-model species [19], [20]. Since 454 sequencing of transcriptomes can produce large numbers of reads with sufficiently long sequences in a short period, comparative transcriptome with 454 sequencing has been performed in many non-model organisms to develop genetic resources and understand molecular mechanisms [21].

In this study, we applied high-throughput 454 sequencing methods to obtain the comparative transcriptome of the hepatopancreas of *P. trituberculatus* during the growth and two ovarian maturation stages, and differentially expressed genes (DEGs) were identified and analyzed. The aims of this study were to enrich the genetic resources for *P. trituberculatus*, and to obtain partial or complete sequences of potential genes involved in nutrition metabolism, ovarian development and other biological activities. A further goal was to identify the patterns of gene expression during growth and ovarian maturation. To our knowledge, this work is the first to report the comparative transcriptome of the hepatopancreas of *P. trituberculatus*. Our results will not only provide valuable genomic information for understanding hepatopancreatic roles in nutrition metabolism and ovarian development of *P. trituberculatus*, but also facilitate further investigations of functional genomics for this species and other closely related species.

## Materials and Methods

### Ethics statement

The wild swimming crabs were captured from East China Sea near Zhoushan Island and no specific permission was required for the sampling area and species because of scientific research purpose. The sampling location is not privately-owned or protected, and the field sampling did not involve endangered or protected species. All handling of crabs were conducted in accordance with guidelines on the care and use of animals for scientific purposes set up by the Institutional Animal Care and Use Committee (IACUC) of Shanghai Ocean University, Shanghai, China.

### Crab culture and dissection

Experimental crabs were caught by fisherman from the East China Sea near Zhoushan Island in mid September 2010 using crab pots. These were transported to hatchery of Dongsheng Aquaculture Company, Zhejiang Province, China. 62 pre-adult females and 20 males were further selected and maintained in a 40 m^2^ concrete tank (Length×Width×Depth = 5 m×8 m×1 m). The identification of pre-adult females was based on the puberty molting as the pre-adult females have the triangular shaped pleonal flaps while the mature female have oval shaped pleonal flaps [15]. On the bottom of the tank, approximately 8–10 cm of sand was provided in 10 m^2^ bottom for the crabs to shelter themselves for the prevention of cannibalism. Throughout the experiment, a static water depth of 70 cm was maintained and 20–30% of water was exchanged daily in the morning for each tank. The water was settled, disinfected by sodium hypochlorite, and then filed by the sand filter. All crabs were fed daily at 16:00 with frozen razor clam *Sinonovacula constricta* at a ration of 3–10% total biomass and adjusted based on daily observations of residual feed. Prior to daily feeding, frozen clam was disinfected by sodium hypochlorite for 15 min while feces and uneaten feed were removed by siphoning. Over the period of the experiment, the tank was gently aerated and subjected to ambient temperature. The water temperature was recorded twice daily (9:00–10:00 and 21:00–22:00, respectively) and fluctuated between 15.3 and 24.3°C. Light was provided by overhead fluorescent ceiling lights set on a photoperiod of 14 h light: 10 h dark and light intensity ranged from 600 to 800 lx. The salinity, pH, dissolved oxygen (DO), ammonia and nitrite levels were monitored regularly and maintained at: salinity: 23–25 g L^−1^; pH: 8.0–9.0; DO: >5 mg L^−1^; ammonia: <0.4 mg L^−1^ and nitrite: <0.05 mg L^−1^, respectively. These water quality parameters were all within the suitable range for *P. trituberculatus* as specified by Xie *et al* [12].

There were three stages of hepatopancreas sampled during the experiments, i.e. hepatopancreas from pre-adult females at inter-molting stage (Hg: hepatopancreas at growth stage), hepatopancreas from adult females at endogenous vitellogenic stage (Hen: hepatopancreas at endogenous vitellogenic stage) and hepatopancreas from adult females at exogenous vitellogenic stage (Hex: hepatopancreas at exogenous vitellogenic stage). The identification of inter-molting stage for pre-adult females was based on Shen *et al* (2011) [22] while the ovarian development cycle was staged according to ovarian histology [15]. After every 20 days, ten to fifteen females were sampled randomly for dissection. Prior to sampling, the crabs were starved 3–4 days to reduce food residual in hepatopancreas. At the pre-puberty molting stage (growth stage), only hepatopancreatic tissues were sampled, while both the hepatopancreas and ovaries were collected at the endogenous and exogenous vitellogenic stages. A small portion of ovaries were also sampled for post-puberty females and fixed in Bouin's solution for the ovarian histology. Based on the ovarian staging results, five replicates were further selected for each stage. In order to prevent the contamination from residual food in the hepatopancreas, the sampled hepatopancreas was rinsed twice with ringer solution to remove the contents in hepatopancreatic tubules, and then stored in liquid nitrogen for the following experiments.

### RNA extraction and mRNA purification

Each frozen sample was ground in a mortar with liquid nitrogen, and then total RNA was isolated using TRIzol reagent (Invitrogen, USA) following the manufacture's protocol. The final total RNA was dissolved in 200 µL RNase-free water. The concentration of total RNA was determined using a NanoDrop2000 spectrophotometer (Thermo Scientific, USA), and the RNA integrity was checked using an RNA 6000 Pico LabChip with the Agilent 2100 Bioanalyzer (Agilent, USA). The total RNA was incubated with 10 U DNase I (Ambion, USA) at 37°C for 1 h, and then nuclease-free water was added to dilute the sample volume to 250 µL. The messenger RNA (mRNA) was further purified with a MicroPoly(A) Purist Kit (Ambion, USA) according to the manufacturer's protocol. The mRNA was dissolved in 100 µL of RNA Storage Solution (Ambion). The final concentration was determined using a NanoDrop2000 spectrophotometer.

### Library construction and sequencing

For each stage of hepatopancreatic or ovarian cDNA library, equal amounts of purified mRNA samples from each replicate were pooled together for the cDNA synthesis. There were a total of five cDNA libraries in this experiment, i.e. hepatopancreas at growth stage (Hg), hepatopancreas at endogenous vitellogenic stage (Hen), hepatopancreas at exogenous vitellogenic stage (Hex), ovary at endogenous vitellogenic stage (Oen) and ovary at exogenous vitellogenic stage (Oex). Double-stranded cDNA was transcribed from mRNA according to Ng's full-length cDNA synthesis protocol with some modifications [23]. GsuI-oligo dT primer was used to the first-strand cDNA synthesis from 10 µg of mRNA using 1000 units of Superscript II reverse transcriptase (Invitrogen, USA). After incubation at 42°C for 1 h, the 5′-CAP structure of mRNA was oxidized by NaIO_4_ (Sigma, USA) and ligated to biotin hydrazide, which was used to select complete mRNA/cDNA heterodimers by binding Dynal M280 beads (Invitrogen). After the second strand cDNA synthesis, the polyA and 5′ adaptor were removed by GsuI digestion.

cDNA size fractionation was performed using a fractionation column (Agencourt, Germany). Each cDNA fraction larger than 800 bp was sonicated to a range of 300–800 bp. The prepared cDNAs were transformed into single-stranded template DNA (sstDNA) libraries by using the GS DNA Library Preparation kit (Roche Applied Science, USA). The sstDNA libraries were clonally amplified in a bead-immobilized form by using the GS emPCR kit and sequenced on a 454 Genome Sequencer FLX instrument (Roche Applied Science).

### EST assembly and functional annotation

The 454 sequencing reads were filtered to remove adaptor sequences, mitochondrial and ribosomal sequences, and low quality reads. The qualified reads from different libraries were pooled together, and then assembled into EST clusters (contigs) using CAP3 software with default parameters. The unassembled reads were considered as singlets. The contigs and singlets were generally referred to as unigenes [20]. Open reading frames (ORFs) of all unigenes were identified with GetORF from EMBOSS package [24]. The ORF of each predicted protein was used for BLASTP searches against the Swiss-prot and Non-redundant protein sequences (nr) databases with an E value of 1e-3, and then the best match was chosen. Gene Ontology (GO) analysis was also performed based on sequence similarity with GoPipe through BLASTP against Swiss-Prot and TrEMBL databases with an E value of 1e-3 [25]. Deduced proteins with homologues in other organisms were used to determine the Clusters of Orthologous Groups (COG) and Kyoto Encyclopedia of Genes and Genomes (KEGG) for the prediction of possible functional classifications and molecular pathways [26]. Because this study was focused on the biological function of the hepatopancreas at the molecular level during the growth and ovarian development, the predicted protein sequences derived from ovary-specific contigs (i.e. assembled with reads only originated from ovary cDNA libraries) and ovary-specific singlets (i.e. reads only originated from ovary cDNA libraries) were removed, and the protein sequences originated from three hepatopancreas transcriptome were used to perform the GO, COG, KEGG and the following analysis. In order to identify the candidate genes involved in food digestion, nutrition metabolism and ovarian development, the unigenes annotated to these physiological functions would be further analyzed. Because of heterogeneity of gene expression, alternative splicing and other technical errors, several unigenes would be annotated to a single gene, therefore, the identification of candidate genes was firstly based on the homogenous search against the Swiss-prot and nr databases. Then, the further analysis was based on the potential function genes, not the unigenes.

### Identification of differentially expressed genes

Reads from different libraries were distinguished and counted from read name in an assembled Ace file. The number of reads for each contig was then transformed into Reads Per kilo bases per Million (RPKM) [27], and differentially expressed genes (DEGs) between the different libraries were identified by the DEGseq package using the MA-plot-based method with Random Sampling model (MARS) method [28]. The false discovery rate (FDR) <0.001 and the absolute value of log_2_Ratio >1 were regarded as the significant difference in gene expression abundance across the libraries.

## Results and Discussion

### 454 sequencing and assembly

To identify the genes involved in nutrition metabolism and ovarian development of *P. trituberculatus*, five cDNA libraries from pooled mRNAs, extracted from the hepatopancreas and ovaries of different developmental stages, were prepared and sequenced using the 454 GS-Flx platform, producing 303,450 raw reads with an average sequence length of 351 bp. The reads produced were used for clustering and *de novo* assembly. After removal of the adaptor low quality (quality<20) mitochondrial and ribosomal sequences, 286,091 clean reads remained. All reads were deposited in the Short Read Archive (SRA) of the National Center for Biotechnology Information (NCBI) with the accession number SRA051608.1. Clustering and assembly of these reads resulted in a non-redundant set of 53,479 unigenes, comprising 21,635 contigs and 31,844 singlets (reads that were not assembled into contig). Among them, most (69.69%) of the contigs' length was distributed in 250–750 bp while 92.28% of the singlets' length was between 200 and 500 bp. This assembly produced a substantial number of large contigs, i.e. 9,148 contigs (42.45%) were more than 500 bp in length, and 8.26% of total contigs (1,788) were more than 1 kb in length. Detailed results of the sequencing, assembly and analysis are shown in Table 1.

**Table 1:** Summary of sequencing results of *Portunus trituberculatus* transcriptome.

Sample	Hg	Oen	Hen	Oex	Hex
Reads assemble in contig	36,155	32,341	69,879	50,062	65,808
Singlets	6,866	6,172	8,585	7,101	3,122
Total contigs	21,635				
Contig average length	465 bp				
Contig length range	46–13,765 bp				
Total singlets	31,844				
Singlet average length	367 bp				
Singlet length range	90–547 bp				

In this study, the mean length of contigs and singlets in *P. trituberculatus* was shorter than that of the oriental river prawn *Macrobrachium nipponense*, i.e. 42,551 contigs and 38,860 singlets with average lengths of 939 bp and 345 bp, respectively [21]. This difference may be related to different tissues or organs used in the library construction for transcriptome sequencing. In *M. nipponense*, the RNA of eyestalk, gill, heart, ovary, testis, hepatopancreas, muscle and different stage embryos were combined for the construction of a cDNA library. It suggested that more tissue combined and a larger number of reads in one cDNA library would lead to a longer mean length of contigs. However, in our study, only the hepatopancreas and ovarian RNA from different stages were used to construct five un-normalized cDNA libraries. Prior to our study (up to July 25, 2013), only 14,372 ESTs were available in public databases for *P. trituberculatus*, and less than 5,000 high quality ESTs were collected in each of the previous *P. trituberculatus* cDNA libraries [17], [18]. Our transcriptome provided a total of 286,091 clean reads for *P. trituberculatus*. Therefore, our study makes a considerable contribution to the genetic resources of *P. trituberculatus*, which will facilitate further molecular investigations on this species.

### Annotation of predicted proteins

Annotation of sequence clusters allows the rapid identification of sequences with similarity to a particular gene of interest, facilitating further studies of closely related genes. In our analysis, open reading frames (ORFs) were found in 21,315 contigs and 27,766 singlets. Further analysis showed that only 7,762 contigs (36.41%) and 4,098 singlets (14.76%) were defined with known biological functions, whereas the remainder required more genetic information for annotation, which is lacking in *P. trituberculatus*. Of those unigenes with ORFs, 11,039 unigenes (7,247 contigs and 3,993 singlets) were matched to known species, while only 2,773 annotated sequences were identified as belonging to crustacean species. As shown in Figure S1, only 209 annotated unigenes matched the registered sequences of *P. trituberculatus* in the GenBank non-redundant database, while the remainder was considered to be newly discovered in this study. This result suggests that by characterizing these unique sequences we have made a significant contribution to the genomic information of *P. trituberculatus*. If a homology search was done for the other crustacean species in the database, there were 1,178 identified unigenes matching to the freshwater cladoceran *Daphnia pulex*, followed by *Scylla paramamosain* (177), *Litopenaeus vannamei* (168), *Eriocheir sinensis* (130), *Portunus pelagicus* (124), *Metacarcinus magister* (110), *Penaeus monodon* (93), *Marsupenaeus japonicus* (72), *Callinectes sapidus* (57) and *Fenneropenaeus chinensis* (54) (Figure S1). Clearly, the largest number of homology genes was matched with *D. pulex*. This is because whole-genome sequencing of *D. pulex* has been completed [29], which has resulted in the large genetic information available in the public database.

A primary target for our transcriptome sequencing projects is the identification of genes involved in nutrition metabolism, ovarian development and other important biological processes. However, for organisms lacking a fully sequenced genome, achieving this goal is hampered by several negative factors [30]. First, only a small percentage of unigenes could be functionally annotated, while a large number of unigenes not matching public databases, remained as separate sequences after gene annotation [21], [31]. This problem was particularly evident in our study owing to the limited genetic information available for *P. trituberculatus*. Second, genes present in the newly sequenced transcriptome are probably mainly represented by fragments, some of which match conserved regions of known genes, while the others, corresponding to the poorly conserved regions (e.g., untranslated regions), are considered to be non-matches. Third, our cDNA libraries were non-normalized, resulting in genes with lower expression levels going undetected. In other cases, sequence fragments may be too short to obtain statistically meaningful matches; there may have been technical issues (e.g., low quality raw data and inaccurate contig assembly), and an inappropriate choice of search parameters (e.g. too stringent for e-value cut-off) could have resulted in no BLAST hits. Furthermore, the methods used to perform the gene annotation may also have omissions. A single sequence could produce several different search results using the various programs and databases, and those results would be considered undetermined. Although the best matches were chosen for the annotation of the sequences in this study, their definite functions still need to be verified by future studies. Therefore, several precautions were undertaken to minimize the influence of technical effects in our study. For example, DNase treatment of RNA was performed before cDNA synthesis to remove genomic DNA contamination, and low quality data was excluded from the assembly process. With the exception of technical issues derived from sequencing, biological factors may be responsible for the large population of sequences without annotation, including genes with lower expression levels, species or tissue-specific genes (present in the studied species, but absent from the databases), rapidly evolved genes (having orthologs in other species, but high divergent genes lead to un-efficient recognition of orthologs), and the persistence of non-coding fractions mainly from untranslated regions of the sampled transcripts [32]. In this study, the first two factors are highlighted since only the hepatopancreas and ovaries were used to construct non-normalized libraries before 454 transcriptome sequencing.

Because this study focused on the biological function of the hepatopancreas at a molecular level during growth and ovarian development, the predicted protein sequences derived from ovary-specific contigs and singlets were removed, and the remaining unique protein sequences, originated from *P. trituberculatus* hepatopancreas transcriptome, were used to perform the following analysis. In three hepatopancreatic transcriptomes, 17,388 contigs and 16,039 singlets had predicted protein sequences, while only 7,954 unigenes (including 5,691 contigs and 2,263 singlets) with the predicted protein sequences were annotated with biological functions (Table S1).The coding sequences homologous to “vitellogenin”, “cryptocyanin”, “hemocyanin”, “endonuclease/reverse transcriptase”, “ribosomal protein”, “reverse transcriptase”, “trypsin”, “zinc finger protein”, “cytochrome P450” and “myosin” were the most abundant, corresponding to active physiological processes in the hepatopancreas (Table S1). Although our study aimed to find putative genes involved in nutrition metabolism and ovarian development, other putative transcripts identified here could also provide a foundation for further understanding hepatopancreatic roles in other biological processes, such as immunity and environmental adaptation. Moreover, these transcriptomic findings could be a good source for deciphering the putative functions of novel genes.

Of those unigenes (including contigs and singlets) with predicted protein sequences, 25,210 (65.44%) unigenes were hepatopancreas-specific. Among them, the number of Hg-specific unigenes was 6,970, while there were 10,467 and 4,597 stage-specific sequences for Hen and Hex, respectively. The lowest number of stage-specific sequences was found in Hex while the highest number of stage-specific sequences was found in Hen. This may be because Hen is a transitional stage from growth to reproduction. At this stage, the growth of the crab does not stop completely, while ovarian development has been initiated [15]. Therefore, more genes are expressed at the Hen stage. Among those Hg-specific sequences, the phenomenon of multi-sequences annotated to a single gene was very rare. Except for conserved hypothetical protein and hypothetical protein, only 69 genes (12.97%) were matched by more than two different sequences in Hg. However, 211 genes (15.69%) were annotated with large numbers of sequences in Hen and Hex, such as vitellogenin (136 unigenes), hemocyanin (64 unigenes), cryptocyanin (53 unigenes), endonuclease/reverse transcriptase (30 unigenes), reverse transcriptase (27 unigenes), trypsin (25 unigenes), zinc finger protein (25 unigenes) and ribosomal protein (20 unigenes) (Table S1). The difference between these two groups might be derived from the more active activities of gene expression and translation performed in the Hen and Hex stages. This is because ovarian development has been initiated soon after crabs entered the Hen stage [3]. Correspondingly, related genes, such as vitellogenin and ovarian development-related protein, started to express at high levels. However, the vitellogenin sequence was not found in the Hg stage. Some other genes, including hemocyanin, cryptocyanin and methyl farnesoate epoxidase, may play additional roles during ovarian development compared with the growth stage. Consequently, they had more abundant sequences matched to their respective genes in the Hen and Hex stages than the Hg stage.

### Annotation of gene ontology (GO) and clusters of orthologous groups (COG)

**Figure 1:**
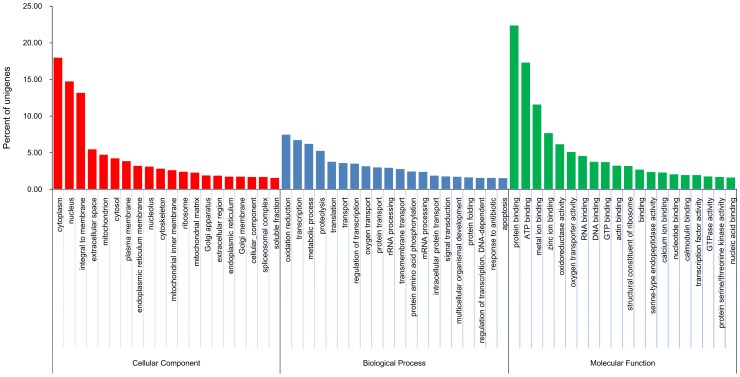
Gene Ontology classification of assembled non-redundant unigenes in the *P. trituberculatus* hepatopancreatic transcriptome. The bar chart shows the percentage of unigenes involved in each main functional terms. Those unigenes are grouped into three categories, including cellular components, biological processes and molecular function, and further categorized into 36 main functional terms.

**Figure 2:**
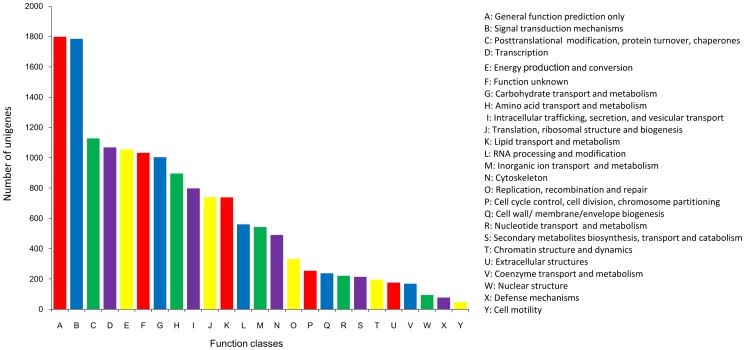
Functional classification of clusters of orthologous groups (COG) of identified unigenes (deduced protein sequences) in the *P. trituberculatus* hepatopancreas transcriptome. The bar chart shows the number of unigenes classified to each major COG.

GO assignment programs were used for functional categorizations of the annotated unigenes. These sequences were grouped into three categories, including cellular components, biological processes and molecular function, and further categorized into 36 main functional terms (Figure 1). A total of 3,523 unigenes were assigned to 3,734 GO terms in our hepatopancreatic transcriptome. Generally, the total number of GO terms obtained from three categories was larger than the total number of the involved unigenes. This is because, in many cases, the same transcript could be assigned to multiple GO terms. Within the cellular components, the highest percentage of unigenes involved in GO terms was the cytoplasm (11.06%), followed by the nucleus (9.36%), and integral to the membrane (7.81%). Of the molecular function categories, the majority of the GO terms were related to protein binding activities (18.70%), ATP binding activities (13.56%), metal ion binding (7.95%), zinc ion binding (5.80%), and RNA binding (3.80%). As for the biological processes, the three major GO terms were oxidation reduction (8.90%), transcription (7.46%) and metabolic processes (5.15%), while the percentages of unigenes involved in other GO terms were less than 5% (Figure 1). Assignments of COG were further used to evaluate the completeness of our transcriptome library and the efficiency of our annotation process. Overall, 9,526 of the deduced protein sequences were matched to the conserved domains database (CDD) of the NCBI, corresponding to 25 COG categories (Figure 2). Of these, the majority of the clusters were “general function prediction only” (1,799, 11.49%), “signal transduction mechanisms” (1,784, 11.40%), “post-translational modification, protein turnover, chaperones” (1,128, 7.21%), “transcription” (1,069, 6.83%), “energy production and conversion” (1,054, 6.73%), “function unknown” (1,033, 6.60%), and “carbohydrate transport and metabolism” (1,003, 6.41%) (Figure 2).

### KEGG pathway analysis

In this study, 1,711 unigenes were predicted in 294 specific pathways, representing six pathway categories, i.e. metabolism, organismal systems, diseases, genetic information processing, environmental information processing and cellular processes (Figure S2). The largest category was metabolism (1,627 unigenes), which included carbohydrate metabolism (413, 25.38%), amino acid metabolism (266, 16.35%), lipid metabolism (215, 13.21%), energy metabolism (169, 10.39%) and other subcategories (Figure S2). These results revealed that the functions of the hepatopancreas were mostly related to nutrition metabolism, which is consistent with its role as an absorption and storage organ [3]. The second largest pathway category was organismal systems (835 unigenes) followed by diseases (676 unigenes) and genetic information processing (675 unigenes). The top three subcategories in each group were the immune system (236), endocrine system (199) and digestive system (148) for organismal systems; immune system diseases (223), cancers (199) and neurodegenerative diseases (189) for diseases; folding, sorting and degradation (263), translation (244) and transcription (92) for genetic information processing (Figure S2), suggesting other biological functions of the hepatopancreas. KEGG pathway analysis is a useful tool for the prediction of potential genes and their functions at a whole transcriptome level. As for the pathway in the different hepatopancreatic stages, 200 Hg-specific sequences were predicted in 167 specific pathways, while 549 sequences in Hen and Hex were classified in 238 pathways (Table S2). The top pathways in these two groups were quite different (Table S2). The predicted pathways, together with the GO and COG analyses, will facilitate the annotation of these unigenes and investigations of gene function in future studies [21].

### Candidate genes involved in food digestion and nutrition metabolism

Although previous research has revealed the functional morphology and cytology, biochemical composition, and digestive enzyme activities of *P. trituberculatus* hepatopancreas [3], [14], [33], very limited molecular information is available for understanding hepatopancreatic nutrition metabolism in this species. Therefore, identification of genes involved in food digestion and nutrition metabolism of the hepatopancreas of *P. trituberculatus* is significant, and would be helpful to understand nutrition metabolism and its physiological process at the molecular level. This study is the first to use transcriptome to discover the potential genes involved in the synthesis and secretion of different digestive enzymes, the absorption and transportation of nutrients, synthesis and decomposition of nutrients for *P. trituberculatus*.

The hepatopancreas has four types of cells, embryonic (E cell), blister-like (B cell), resorptive (R cell) and fibrillar (F cell) [3]. It is generally accepted that F cells only synthesize and secrete zymogen, which is then discharged into the hepatopancreatic lumen, and is eventually accumulated and stored in the cardiac stomach in an active form [1]. In this study, 49 unigenes were classified in the digestive system, which was composed of pancreatic secretion (41), protein digestion and absorption (34), salivary secretion (17), carbohydrate digestion and absorption (14), and gastric acid secretion (10) (Table S3). We identified seventeen genes related to the biosynthesis or modification of different digestive enzymes, including five genes for protein digestion, six genes for carbohydrate digestion, and six genes for lipid digestion. Five protease genes included trypsin, chymotrypsin, trypsin-like serine proteinase, carboxypeptidase B, and metalloproteinase exist in the hepatopancreas of *P. trituberculatus* (Table 2). Previous studies have demonstrated that there are four types of proteases, including trypsin, chymotrypsin, carboxypeptidase and metalloproteinase (*Astacus* protease), in the freshwater crayfish *Astacus astacus* and penaeid shrimp *Penaeus semisulcatus* [1], [34]. Both trypsin and chymotrypsin belong to serine proteases, but they have a peptide chain preference for protein hydrolysis. Trypsin cleaves peptide chains mainly at the carboxyl side of the amino acids for lysine or arginine, while chymotrypsin prefers the peptide chains of aromatic amino acids such as tryptophan, phenylalanine and tyrosine. Moreover, trypsin is also involved in the processing of other digestive enzymes like the activation of proenzymes, such as chymotrypsinogen, proelastase and procarboxypeptidase [35]. Carboxypeptidase is one of the exopeptidases that cleave the peptide bond of an amino acid residue at the carboxy-terminal end, leading to the release of free amino acids and further participating in the processing of peptides and proteins [36]. Metalloproteinase is one family of protease enzymes whose catalytic mechanism involves a metal, such as zinc and cobalt [37].

**Table 2:** Candidate genes involved into nutrient digestion and transport in the hepatopancreas of *P. trituberculatus*.

Functional category	Gene	Hits	Length (bp)	Number of reads in each stage		
				Hg	Hen	Hex
Protein digestion	trypsin	44	110–1509	656	1875	1087
	trypsin-like serine protease	3	357–1225	258	726	205
	chymotrypsin	2	389–844	4	22	0
	carboxypeptidase B	6	343–1982	344	438	402
	metalloproteinase	1	985	0	20	1
Lipid digestion	triacylglycerol lipase	3	384–1426	10	61	11
	pancreatic lipase-related protein 2	2	431–1764	2	39	0
	phospholipase C	2	413–465	0	7	4
	phospholipase D	3	405–748	3	1	3
	lipase maturation factor 2	1	820	5	0	3
Carbohydrate digestion	preamylase 1	2	257–2918	47	41	38
	β-N-acetylglucosaminidase	3	412–2238	3	35	6
	chitinase	18	238–2361	218	951	199
	chitinase precursor	15	226–2292	119	732	56
Nutrient transport	low-density lipoprotein receptor	7	350–511	2	10	58
	high density lipoprotein binding protein	2	366–1597	4	12	8

The sequence IDs for each gene and more details were shown in Table S3.

Six candidate genes for lipid digestion were identified in the hepatopancreas of *P. trituberculatus*, including triacylglycerol lipase, pancreatic lipase-related protein 2, phospholipase C, phospholipase D, lipase maturation factor 2, and lipase maturation factor 2 (Table 2, Table S3). Triacylglycerol lipases, generally named lipases, play an important physiological role, and are involved in triacylglyceride catabolism of crustaceans and mammals [38], [39]. Triacylglycerol lipases can be divided into two classes, i.e. digestive and intracellular lipases. Digestive lipase is exclusively expressed in the hepatopancreas, suggesting that they function as a digestive enzyme, while intracellular lipase was found in the pleopods, digestive tube, uropods, hemocytes, muscle and gonad, indicating it functions in the mobilization of energy reserves [39]. Because of higher mRNA expression levels of triacylglycerol lipases in *P. trituberculatus* hepatopancreas, it is suggested that this triacylglycerol lipase is likely a digestive lipase for *P. trituberculatus*. In mammals, lipase maturation factor (Lmf) is critical for the post-translational activation of three vascular lipases: lipoprotein lipase, digestive lipase and endothelial lipase, which is likely involved in the assembly of inactive lipase monomers into active dimmers and/or the stabilization and maintenance of the homodimer structure [40]. A similar mechanism may exist in crustaceans since Lmf1 and Lmf2 transcripts were found in *P. trituberculatus* hepatopancreas. A phospholipase is an enzyme that hydrolyzes phospholipids into fatty acids and other lipophilic substances. There are four major classes, termed A, B, C and D, distinguished by the type of reaction they catalyze. Only phospholipase C and phospholipase D genes were identified from the hepatopancreas of *P. trituberculatus* in this study.

For carbohydrate digestion, amylase and phosphorylase are very important for the cleavage of starch and glucogen [4], [41]. There were two preamylase transcripts detected in our cDNA libraries of *P. trituberculatus* hepatopancreas (Table 2), and the expression level was higher at the Hg stage compared with the Hen and Hex stages. This maybe because the digestion of carbohydrates mainly happens at the Hg stage, whereas lipids are the major digestive nutrient at the Hen and Hex stages. This was confirmed by the large number of unigenes classed into lipid metabolism in our study. Chitinase and its related transcripts, together with β -N-acetylglucosaminidase transcript, which are considered to participate in the digestion of chitin, were detected in the cDNA libraries (Table 2). Hepatopancreatic chitinases and chitin metabolism-related genes not only function as digestive enzymes for ingested chitin, but are also involved in the digestion of endogenic chitin, such as stomach chitin, during the premolt [42], [43].

For lipid transport in plasma, triglycerides and cholesterol esters are packaged into lipoprotein particles in which they form a hydrophobic core surrounded by a surface monolayer of polar phospholipids [44]. Lipoproteins include very low density lipoprotein (VLDL), low density lipoprotein (LDL), intermediate density lipoprotein (IDL) and high density lipoprotein (HDL), and very high density lipoprotein (VHDL) [45], [46]. Chylomicrons and VLDL are undoubtedly lipoproteins that carry triglycerides from sites of absorption and synthesis to storage and utilization sites. In our study, seven transcripts of LDL receptor (Table 2) and one transcript of very low density lipoprotein receptor (Table S3) were identified. These LDL receptors can remove cholesterol-rich intermediate IDL and LDL from plasma and thereby regulate plasma cholesterol levels [45]. The main protein fraction of crustacean ovarian proteins is HDL, frequently associated with carotenoids and usually referred to as lipovitellin [46]. Unfortunately, no HDLs were found in our study, but two transcripts of high density lipoprotein (HDL) binding protein were identified (Table 2). The HDL binding protein might be involved in the recognition or transportation of HDL.

As mentioned, some of the absorbed nutrients will be transported to the gonads, muscle and other tissues from the hepatopancreas during the reproduction and growth process, while the hepatopancreas is regarded as a major site for nutrient catabolism and anabolism [3], [4]. Protein is the first important nutrient for animal growth and reproduction, and has many functions, such as an energy source and structural material of the cell membrane. In this study, there were 392 unigenes involved in protein metabolic pathways, and 194 unigenes related to amino acid metabolism. Five amino acid synthetase transcripts with higher expression levels were identified in our study (Table 3). Generally, glutamine synthetase participates in glutamine synthesis from L-glutamic acid, which can be further transformed to most of the non-essential amino acids needed. Glutamate synthase is mainly involved in the synthesis of glutamic acid from its precursor α-ketoglutarate [47]. Glutamate dehydrogenase can synthesize α-ketoglutarate from glutamine by dehydrogenation, while the product will be converted to alanine by de-amination [48]. D-3-phosphoglycerate dehydrogenase is an important enzyme in the upstream pathway of serine biosynthesis, by synthesizing 3-phosphate hydroxyl acetone acid from 3-phosphoglycerate [49]. Phosphatase is responsible for hydrolyzing phosphor-amino acids and releasing amino acid and phosphoric acid, which is the last step for the synthesis of many amino acids [50]. There are also several unigenes involved in amino acid decomposition, including alanine aminotransferase, γ-glutamyltranspeptidase 1 and glutamate dehydrogenase (Table 3).

**Table 3:** Candidate genes involved into nutrition metabolism in the hepatopancreas of *P. trituberculatus*(protein and lipid metabolism).

Functional category	Gene	Hits	Length (bp)	Number of reads in each stage		
				Hg	Hen	Hex
Protein metabolism	glutamine synthetase	4	457–1697	11	98	10
	glutamate synthase	5	410–1156	3	11	7
	glutamate dehydrogenase	2	452–2224	3	23	6
	D-3-phosphoglycerate dehydrogenase	2	431–2088	12	0	31
	phosphatase	5	265–963	1	9	44
	alanine aminotransferase	10	169–1052	8	9	4
	γ-glutamyltranspeptidase 1	1	993	1	4	3
Lipid metabolism	acyl-CoA oxidase	2	649–841	1	4	1
	conserved hypothetical protein(related to very-long-chain acyl-CoA dehydrogenase)	2	345–579	0	6	0
	acyl-CoA dehydrogenase isoform 1	1	1253	3	5	1
	alcohol dehydrogenase	4	403–2129	36	31	59
	mitochondrial-like D-β-hydroxybutyrate dehydrogenase	1	784	1	4	0
	long-chain fatty acid CoA ligase 4	2	407–1218	4	15	5
	prostaglandin D2 synthase, hematopoietic-like	1	1322	52	25	15
	prostaglandin E synthase 2	1	1062	2	3	0
	cytochrome P450 CYP379A1	17	298–1928	33	87	114
	fatty acid binding protein 1	4	315–961	42	32	14
	intracellular fatty acid binding protein	2	475–2011	43	74	48
	fatty acid synthase	7	295–1552	6	0	34
	conserved hypothetical protein(related toΔ6-fatty acid desaturase/Δ-8 sphingolipid desaturase)	2	1044–2585	18	56	22
	conserved hypothetical protein(related to fatty acid elongation in mitochondria)	5	611–746	6	12	5
Carbohydrate metabolism	glucosyl/glucuronosyl transferases	3	458–1181	6	12	53
	chitin synthase B	1	1581	2	11	2
	UDP-glucuronosyltransferase 2B14-like	2	454–722	9	5	1
	glucosamine-6-phosphate isomerase	1	432	1	1	4
	fructose 1,6-bisphosphatase	2	354–1988	40	28	21
	phosphoglycerate kinase	2	791–1088	7	12	18
	glyceraldehyde 3-phosphate dehydrogenase	5	353–1514	44	67	78

The sequence IDs for each gene and more details were shown in Table S3.

In this study, there were 142 unigenes identified that are involved in lipid metabolism pathways (Table S3). Glycerophospholipid metabolism took the biggest proportion, which had 34 unigenes mapped, followed by fatty acid metabolism (29), sphingolipid metabolism (26), glycerolipid metabolism (23), arachidonic acid metabolism (15) and biosynthesis of unsaturated fatty acids (14). The hepatopancreas is generally considered a major lipid storage organ and plays an important role in lipid metabolism in crustaceans [51]. In crustaceans, neutral lipids serve as the major energy source, and triacylglycerol (TAG) is the major storage lipid in the hepatopancreas, which supports the energy needs for key physiological functions such as moving, metamorphosis and reproduction [3]. A previous study has revealed that substrate specificity of lipase and fatty acid composition of TAG may be an important factor to determine which fatty acids are mobilized during lipolysis for oxidation in crustaceans [52]. There was one acyl-CoA oxidase that exists in the hepatopancreas of *P. trituberculatus* (Table 3), which is necessary for the oxidation of fatty acids, generating aldehydes and ketones. The first reaction of mitochondrial β-oxidation is catalyzed by acyl-CoA dehydrogenases. There are four kinds of acyl-CoA dehydrogenases in animal mitochondrial β-oxidation, i.e. very-long-chain acyl-CoA dehydrogenase (VLCAD, having more than 24 carbons), long-chain acyl-CoA dehydrogenase (LCAD, acyl chain length of 14–22 carbons), medium-chain acyl-CoA dehydrogenase (MCAD, acyl chain length of 6–12 carbons) and short-chain acyl-CoA dehydrogenase (SCAD, acyl chain length of less than 6 carbons). VLCAD is responsible for the catalysis of dehydrogenasing the very long-chain fatty acids in the first step of mitochondrial fatty acid oxidation [53]. In our study, two VLCAD and one acyl-CoA dehydrogenase isoform 1 were identified in the hepatopancreas of *P. trituberculatus* (Table 3). Moreover, there were three kinds of other dehydrogenase transcripts in the hepatopancreas, i.e., fouralcohol dehydrogenases, a mitochondrial-like D-β-hydroxybutyrate dehydrogenase (Table 3), and a short-chain dehydrogenase (Table S3). The metabolism of arachidonic acid (ARA, 20: 4n6) is very complicated, because it is involved in β-oxidation for energy, and serves as a precursor for eicosanoids, an important class of lipid mediators [54]. In the cytoplasm, free fatty acids were rapidly converted to fatty acyl-CoA esters by fatty acid-CoA ligase (FACL), and ARA was preferentially converted to ARA-CoA esters by FACL4. Therefore, FACL4 plays an important role in ARA metabolism [55]. FACL4 unigene was also discovered in our study (Table 3), which was also classified into the peroxisome proliferator activated receptor (PPARs) signaling pathway, and participated in the endocrine system, suggesting other potential functions except for fatty acid metabolism. For the metabolism of ARA to eicosanoids, cyclooxygenase is the first key enzyme to catalyze the conversion of ARA to PGH2. PGH2 is subsequently converted to PGE2, PGD2, PGF2a, and PGI2 by specific prostaglandin synthases [56]. Correspondingly, there were two prostaglandin synthase transcripts and seventeen cytochrome P450 CYP379A1 unigenes identified in this study (Table 3). All of these unigenes are involved in prostaglandin metabolism [54]. Fatty acid binding proteins (FABPs) are the most abundant proteins in the cytosol of cells, and are most active in uptake, metabolism, oxidation and storage of long chain fatty acids (LCFA) [57]. Previous studies suggested that hepatopancreas-mediated lipid transport occurs during rapid ovarian development in *E. sinensis* [58], [59]. In our study, four FABPs, two intracellular FABPs transcripts (Table 3) and one sterol carrier protein 2 transcript (Table S3) were found in the hepatopancreas of *P. trituberculatus*.

For lipid synthesis, there were 26 unigenes identified in the hepatopancreas of *P. trituberculatus* (Table S3). Of them, three gene families are involved in fatty acid synthesis, nominating fatty acid synthase (FAS), fatty acid desaturase (FAD) and fatty acid elongase (FAE) [54]. In our transcriptome, all of their transcripts were found and there were 2–7 unigenes matched to them (Table 3). It is worth noting that palmitate (C16:0), a major hepatopancreatic fatty acid in crustaceans, is one of the products of FAS [60]. Both myristate (C14:0), and palmitate (C16:0) serve as substrates for chain elongation to produce long chain fatty acids (LCFAs) via FAD and FAE [61]. Furthermore, there were two unigenes related to the biosynthesis of phosphatidylcholine and phosphatidylethanolamine (contig21267 and contig8716), and one unigene (contig11528) related to synthesis of triacylglycerol (Table S3). These results highlight the importance of the hepatopancreas in lipid metabolism for *P. trituberculatus*. In addition, our transcriptome also revealed several unigenes related to the metabolism of carbohydrates and vitamins (Table 3, Table S3).

### Candidate gene involved in ovarian development

Ovarian development is a very important physiological process for crustacean reproduction. Generally, pond-reared female *P. trituberculatus* have poor ovarian maturation, leading to inferior reproductive performance [11]. Therefore, the identification of genes related to ovarian maturation is significant and would be helpful for understanding the molecular mechanism of ovarian development as well as artificial propagation. Our previous study has shown that the hepatopancreas of *P. trituberculatus* is the major site for the exogenous vitellogenesis stage [16]. In our transcriptome, there were 143 distinct vitellogenin transcripts identified in the hepatopancreas. The length of the unigenes ranged from 149 to 9,503 bp (Table 4), and all of them were matched to one fragment of full-length vitellogenin (Vg) cDNA [8]. These transcripts were most likely the result of alternative splicing and/or alternative expression of the Vg gene for *P. trituberculatus*. A similar phenomenon was found in the red crab, *Charybdis feriatus* [62]. Six transcripts of vitelline membrane outer layer 1-like protein (VMO1) were also found in the hepatopancreas of *P. trituberculatus* (Table 4). In crustaceans, VMO1 proteins are synthesized in the hepatopancreas and are then transported via the hemolymph to developing oocytes. The major role of the vitelline membrane is to avoid mixing of yolk and albumen [63]. In *Daphnia magna*, expression of VMO1 seems to precede the Vg gene, possibly revealing important functional insights into the timing of *D. magna* oogenesis [64].

**Table 4:** Candidate genes involved into ovarian development, immunity and other important biological processes in the hepatopancreas of *P. trituberculatus*.

Functional category	Gene	Hits	Length (bp)	Number of reads in each stage		
				Hg	Hen	Hex
Vitellogenesis	vitellogenin	143	149–9503	0	54	11287
	vitelline membrane outer layer 1-like protein	6	378–898	95	167	110
Reproductive endocrine	cytochrome P450 3A41	1	2894	45	86	119
	24-dehydrocholesterol reductase	1	746	0	1	4
	ecdysteroid-regulated protein	6	436–907	5	25	9
	farnesyl pyrophosphate synthase	1	1552	1	3	1
	farnesoic acid O-methyltransferase	3	673–1225	34	7	47
	JHE-like carboxylesterase 1	6	363–2484	41	76	36
	JHE-like carboxylesterase 2	6	378–656	1	25	4
Immunity and other processes	hemocyanin	8	317–912	6	78	9
	hemocyanin subunit	89	112–5605	825	5628	1877
	calreticulin precursor	1	1763	6	6	56
	ferritin 1	3	392–731	40	39	25
	ferritin 2	2	414–1711	25	58	47
	ferritin 3	3	311–1470	141	430	139
	C-type lectin receptor	2	688–969	49	18	3
	cathepsin L	4	409–1533	88	74	179
	cathepsin C	5	402–1884	45	27	23
	peritrophin-44	2	756–1020	28	13	33
	chymotrypsin BI	5	392–1069	211	395	220
	chymotrypsin BII	8	471–1510	885	1640	821
	β-1,3-glucan-binding protein	11	319–8051	273	429	142
	cryptocyanin	32	100–3373	5152	5	6885
	cryptocyanin 1	74	112–1169	232	26	496
	cryptocyanin 2	30	171–1210	154	1	341

The sequence IDs for each gene and more details were shown in Table S3.

Ovarian development is modulated by a variety of hormones including sex steroids. For example vitellogenin synthesis in the hepatopancreas is induced by estradiol and progesterone [65]. Previous studies have shown that the crustacean hepatopancreas is an important site for steroid hormone biosynthesis and catabolism [7], [66], [67]. Furthermore, some key enzymes for steroid hormone biosynthesis were found in the hepatopancreas of crustacean [7], [68]. In our hepatopancreatic transcriptome, three unigenes were identified for steroid hormone biosynthesis, including cytochrome P450 3A41 (Table 4), 3-hydroxysteroid dehydrogenase (3-HSD), and short-chain dehydrogenase (SDR) (Table S3). 3-HSD is one key family of enzymes that catalyzes the reduction/oxidation of the keto/hydroxyl group at the 3-position of the steroid backbone [68].

It was noteworthy that, steroid hormones, such as estrogen and progesterone, are all products transformed from cholesterol by various enzymes. Therefore, the biosynthesis and metabolism of cholesterol may also have certain effects on ovarian development regulated by these steroid hormones [68]. Here, we identified and classified the genes involved in cholesterol metabolism, including one sequence matched to sterol carrier protein 2 (SCP-2) (Table S3), a 24-dehydrocholesterol reductase (DHCR24) gene (Table 4), and a putative 3 hydroxysteroid dehydrogenase gene (Table S3). SCP-2 is a basic protein with multiple roles not only involved in isoprenoid and cholesterol metabolism [69], but also in cholesterol transport through the cytoplasm [70]. Moreover, DHCR24 is anchored to the membrane of the endoplasmic reticulum and catalyzes the conversion of desmosterol to cholesterol, which is the last step of cholesterol biosynthesis [71].

In decapod crustaceans, the Y-organ (YO) is the primary site for ecdysteroid synthesis while the hepatopancreas is the major site for the inactivation of ecdysteroids. Inactivation involves the conversion of active ecdysteroids to more polar metabolites and/or formation of conjugates. In decapods, the two major organs responsible for removing ecdysteroids from the hemolymph are the antennal gland and hepatopancreas [72]. In general, the antennal gland and peripheral tissues are responsible for the conversion of ecdysteroids to inactive polar metabolites, which are then excreted in the urine. The hepatopancreas is the primary site for the production of apolar conjugates (perhaps fatty acyl esters), which are then eliminated in the feces [73]. Although the hepatopancreas can excrete hemolymph ecdysteroids and their metabolites, it functions primarily in sequestering ecdysteroids obtained from the diet to prevent disruption of the ecdysteroid-regulated processes. Therefore, the hepatopancreas plays an important role in ecdysteroid metabolism and maintaining ecdysteroids at a proper level [74]. However, except for DHCR24 mentioned above, only six transcripts matched to ecdysteroid-regulated protein (Table 4), and a transcript annotated with ecdysone inducible protein 75 (Table S3) were found in our hepatopancreatic transcriptome. These genes might be involved in ecdysteroid metabolism and related activities.

Methyl farnesoate (MF), a crustacean juvenile hormone (JH) analog, is secreted by the mandibular organ (MO) in crustaceans [75]. Previous studies suggest that MF is involved in the regulation of crustacean reproduction and molting [76]. The biosynthetic pathway of MF is divided into an early and a late step [77]. In the early step, farnesyl pyrophosphate (FPP) is produced by the classical mevalonate pathway, which is common to vertebrates and invertebrates [78]. The late step, that is unique to arthropods, is when FPP is hydrolyzed by a pyrophosphatase to farnesol, then oxidized to farnesal and farnesoic acid (FA) by an alcohol dehydrogenase and an aldehyde dehydrogenase, respectively [77]. Finally, FA is converted by farnesoic acid O-methyltransferase (FAO-MeT) in the presence of S-adenosyl methionine (SAM) and a P450 monooxygenase to form MF [79]. The sequences related to MF synthesis were FPP synthase and FAO-MeT in our transcriptome (Table 4). Interestingly, FAO-MeT activity has only been detected in the MO, but its transcripts were also found in the hepatopancreas, gonad, brain, heart, eyestalk, epidermis and gill of some crustaceans [80]–[82]. Therefore, the FAO-MeT gene expression in multiple tissues is an open question for the regulation mechanism of MF during crustacean reproduction.

Although the synthetic pathway of MF is well established, little is known about its degradation in crustaceans. In insects, juvenile hormone esterase (JHE), a carboxylesterase, is responsible for JH inactivation. In crustaceans, catabolism of MF may occur through ester hydrolysis by the specific carboxylesterases (MF esterase) because MF esterase activity was found in the hepatopancreas and reproductive organs in some species [75]. Therefore, the hepatopancreas and gonad appear to be the major sites for MF inactivation [83]. Recently, two JHE like carboxylesterase cDNAs were cloned from the shrimp, *Pandalopsis japonica* [84]. Two JHE like carboxylesterases were identified in our transcriptome (Table 4). Therefore, *P. trituberculatus* hepatopancreas is very involved in ovarian development and has a significant contribution to ovarian maturation.

### Candidate gene involved in immunity

There are several genes identified in our transcriptome, which are special for crustaceans. One group is hemocyanin, a large copper-containing protein family present in the hemolymph of both mollusks and arthropods [85]. In many crustaceans, hemocyanin acts as an antiviral agent against a variety of DNA and RNA viruses [86], [87]. In this study, 97 different sequences were matched to hemocyanin and its subunits (Table 4). These homologous sequences were classified into six pathways, indicating the multi-functions of hemocyanin. Other unigenes involved in immune defense including the chitinase family, trypsin, pancreatic lipase-related protein 2-like (Table 2) and ferritin proteins, C-type lectin receptor, cathepsin L, cathepsin C, peritrophin-44, chymotrypsin B I/II, and β-1,3-glucan-binding protein (Table 4). Their potential roles in the hepatopancreas will be explained and discussed in the following section.

### Differentially expressed genes (DEGs) in different developmental stages

**Figure 3:**
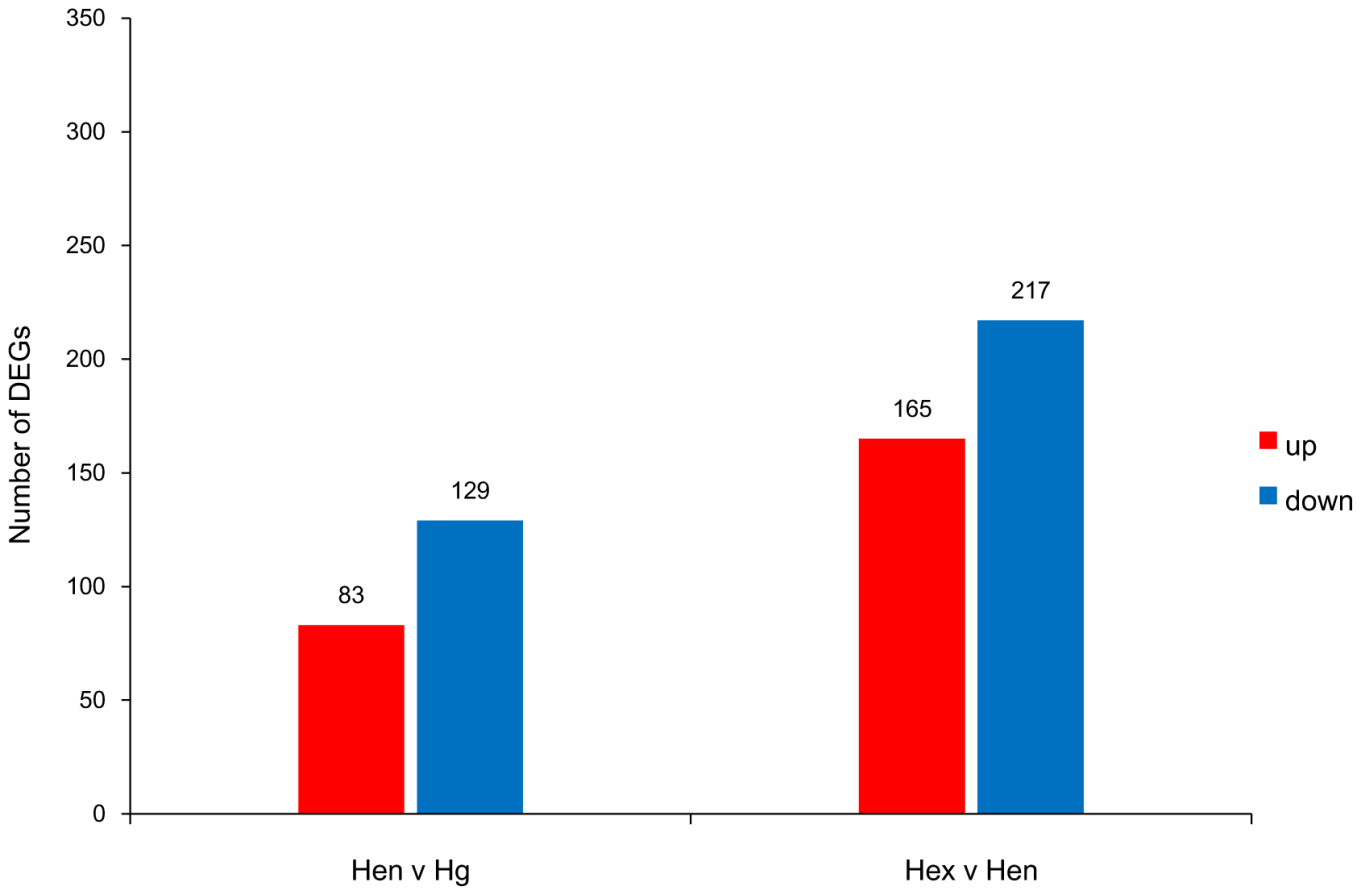
Changes in gene expression profile among different stage hepatopancreas of *P. trituberculatus*. The number of up-regulated and down-regulated genes between Hen and Hg, or Hex and Hen are shown on the top of bars.

To identify DEGs potentially involved in the function of the hepatopancreas, three non-normalized cDNA libraries of the hepatopancreas from different physiological stages were constructed to perform 454 transcriptome sequencing. Therefore, genes with higher expression levels would have a greater chance of being detected, and the number of reads for a given sequence could reflect the relative mRNA expression level in those samples [88]. There were 212 DEGs detected between Hen and Hg, including 83 up-regulated genes and 129 down-regulated genes in Hen compared with Hg (Figure 3). As for Hex compared with Hen, there were 382 DEGs detected, including 165 up-regulated genes and 217 down-regulated genes (Figure 3). In total, there were 431 DEGs detected among the three stages of the hepatopancreas. To understand the functions of these DEGs, all DEGs were assigned to 127 different pathways through the KEGG database. These 127 pathways were grouped further into 33 different classes (Table S4). Among these, amino acid metabolism took the largest proportion, followed by translation, carbohydrate metabolism and biosynthesis of other secondary metabolites which suggests strong nutrition metabolism in the hepatopancreas and its involvement in the growth and ovarian development of *P. trituberculatus* (Table S4).

In the comparison between Hen and Hg, up-regulated genes were related to amino acid metabolism (nine unigenes), translation (six unigenes), digestive system (six unigenes), carbohydrate metabolism (five unigenes), endocrine system (four unigenes), signal transduction (three unigenes) and lipid metabolism (one unigene) (Table S4). Among these up-regulated genes, three proteases, three amino acid metabolism enzymes, one lipase (pancreatic lipase-related protein 2-like), chitinase, and chitinase 1 precursor were found to have higher expression levels in Hen than in Hg (Table S4, S5). Previous studies have shown that the hepatopancreas during ovarian development of *P. trituberculatus* had higher activity of lipase and protease than at the growth stage [33]. This is consistent with our current finding. The higher transcripts of digestive enzymes in Hen may suggest a higher nutrition requirement of protein and lipid during early ovarian development than the growth stage for *P. trituberculatus* [3]. Although the vitellin/vitellogenin is mainly synthesized by the ovaries during the endogenous vitellogenic stage, the nutrients, i.e., protein, lipid and pigments, are absorbed and transported by the hepatopancreas [15]. Therefore, higher levels of protease and lipase transcripts were detected in Hen than in Hg (Table S5). Generally, chitinase is involved in the digestion of endogenic chitin during the premolt stage [43]. Although there was no molting of *P. trituberculatus* females during ovarian development, chitinase and chitinase 1 precursors were up-regulated in Hen (Table S5). This could be explained by the immune function of chitinase [89]. Hemocyanin (Hc) is mainly synthesized by the hepatopancreas [90]. Recent studies have revealed that in crustaceans, Hc is not only an oxygen transporter, but also related to energy storage, exoskeletal sclerotization, osmotic regulation during the molting process, and the innate immune defense [86], [91], [92]. Three up-regulated Hc genes, including hemocyanin subunits, hemocyanin subunit 1 and hemocyanin subunit 2, were found in Hen compared with Hg (Table S5), which suggests that Hen has a greater energy metabolism, oxygen consumption, and immunity than Hg. Ferritin, a major iron storage protein of most living organisms, plays a crucial role in iron metabolism and immunity [93], [94]. Both ferritin 2 and 3 were up-regulated in Hen (Table S5), indicating their important role in this stage. For translational regulation, there were six up-regulated unigenes in *P. trituberculatus* Hen (Table S4, S5). These were involved in many translational regulation steps, such as cell proliferation and RNA transport [95]. These up-regulated transcripts also indicated Hen had stronger general gene translation and expression than Hg.

The down-regulated genes of Hen and Hg mainly consisted of the cryptocyanin family, C-type lectin receptor, cathepsin L, cathepsin C, peritrophin-44, preamylase 1, prostaglandin D2 synthase, and nucleotide excision repair protein (Table S5). First, the cryptocyanin family and C-type lectin receptor are related to crustacean molting [96], [97]. Cryptocyanin, which is closely related structurally and phylogenetically to arthropod hemocyanin, lacks several of the six critical copper binding histidines, and has lost the ability to bind to oxygen. In the Dungeness crab *Cancer magister*, both cryptocyanin and Hc are specifically expressed in the resorptive cells of the hepatopancreas and secreted into the hemolymph. However, cryptocyanin plays a major role in forming the new exoskeleton, while hemocyanin functions in oxygen transport [96]. In our study, there were three down-regulated cryptocyanin subunit unigenes in Hen, which is evident by very low expression levels or no expression of their mRNA (Table4). The changes of a cryptocyanin expression pattern may explain why no molting occurs during the endogenous vitellogenic stage of *P. trituberculatus*, and no new exoskeleton forms during this stage [15]. Therefore, no cryptocyanin is required for the formation of new exoskeleton during the endogenous vitellogenic stage of *P. trituberculatus*. A recent study has proposed that the C-type lectin receptor can recognize and attach to endogenous glycoproteins in the cuticular matrix based on their glycosylation patterns, facilitating the inhibition of calcification during the pre- and post-molting stage of *P. pelagicus* [97]. During ovarian development of *P. trituberculatus*, the exoskeleton is calcified and hardened, leading to the lower expression levels of C-type lectin receptor mRNA in Hen than that in Hg. As for peritrophin-44, cathepsin L and C, all of these are immune proteins [98]. However, it is unclear why these unigenes were down-regulated in Hen. Prostaglandin D2, one of the 2-series prostaglandins, originated from ARA (20:4n-6), which has a very limited physiological function in crustacean ovarian development [99]. This is supported by the lower concentration of prostaglandin D2 in the ovaries and hepatopancreas of *O.senex senex*. It is reasonable to conclude that prostaglandin D2 synthase transcript is down-regulated in Hen with an endogenous vitellogenic stage (Table S5).

In a comparison between Hex and Hen, up-regulated genes mainly included vitellogenin, purple acid phosphatase, ATP binding cassette transmembrane transporter, FAO-MeT, cryptocyanin, hemocyanin subunit 4, cathepsin L, and bax inhibitor-1-like protein (Table S5). During the exogenous vitellogenesis stage of *P. trituberculatus*, the hepatopancreas is the principle site for vitellogenesis, which is supported by the highest Vg mRNA levels in Hex [16]. Purple acid phosphatases (PAPs) belong to the family of dinuclear metallohydrolases, which require two closely spaced metal ions forming a dinuclear center to carry out a hydrolytic reaction [100]. PAP is also an efficient ATPase, and it has been speculated that this enzyme plays a crucial role in the ATP-dependent regulation of bone calcium homeostasis [101]. ATP-binding cassette transmembrane transporters (ABC transporters) are large, membrane-bound proteins, present in all living organisms from prokaryotes to mammals. Their function has been identified as mediating the transport of a variety of substrates, including amino acids, lipids, inorganic ions, peptides, metals, drugs, and proteins across cellular membranes [102]. Our results showed both PAPs and ABC transporters are dramatically up-regulated in Hex, indicating a high nutrient requirement during the exogenous vitellogenesis stage [15]. Therefore, high expression levels of PAPs and ABC transporter transcripts may facilitate nutrient absorption and transport during the exogenous vitellogenesis stage of *P. trituberculatus*. In the shrimp *Metapenaeus ensis*, cathepsin L mRNA only exists in fibrillar cells of the hepatopancreas, while cathepsin L is localized in the blister-like cells, functioning in intracellular and extracellular digestion [103]. In our study, four distinct transcripts, corresponding to cathepsin L, displayed an up-regulated pattern in Hex, which also suggests strong food digestion and nutrient absorption occurring in Hex to support the developing ovaries of *P. trituberculatus*. As mentioned above, in crustaceans, cryptocyanin generally plays an important role in the formation of the new exoskeleton and therefore, cryptocyanin and its mRNA is not detectable in the hemolymph or hepatopancreas of *C. magister* [96]. In our transcriptome, five distinct transcripts, corresponding to cryptocyanin, and cryptocyanin subunit 1 and 2 (Table S5), displayed significantly up-regulated patterns in Hex, which may participate in fast ovarian development of *P. trituberculatus*. During the early exogenous vitellogenesis stage of *P. trituberculatus*, the mandibular organ (MO) has the highest biosynthetic ability of Methyl farnesoate (MF), leading to the highest MF titer in the hemolymph [104]. FAO-MeT is one of the key enzymes for the conversion of JH to MF [75]. Our transcriptome showed that FAO-MeT transcripts were significantly up-regulated in Hex (Table S5).

In Hex, major down-regulated genes consisted of the hemocyanin family, chitinase family, trypsin, pancreatic lipase-related protein 2-like, chymotrypsin B-II, β-1,3-glucan-binding protein, triacylglycerol lipase, zinc proteinase Mpc1, ferritin 3, map kinase-interacting serine/threonine, ribosomal protein family, and elongation factor 1 and 2 (Table S5). Most of these could be divided into two categories; I) related to immune functions (16 unigenes), and II) related to translation (20 unigenes) (Table S5). Reproduction and immunity are both important to organismal fitness. Although production of offspring is an essential fitness component, an organism must survive and maintain sufficient health to reproduce [105], [106]. Because both reproduction and immunity require large resource investments, optimizing one process often comes at a cost to the other [107]. Therefore, trade-offs are taken as evidence of conflicts among reproduction and immunity [108]. During the exogenous vitellogenesis stage of *P. trituberculatus*, females are investing large amounts of energy and nutrients into the synthesis of vitellogenin in the hepatopancreas, which is deposited into the developing ovaries [15]. This process is supported by the very high expression levels of Vg mRNA in Hex [16]. The fast synthesis of vitellogenin in the hepatopancreas may lead to decreasing resources available to the immune system, thereby down-regulating the immune-related unigenes. It is important to understand the mechanisms of competition and trade-offs between ovarian development and the immune function of the swimming crab *P. trituberculatus*. Because many immune-related unigenes are down-regulated in Hex, the genes related to translation were also coincidently decreasing in their expression levels in our transcriptomes, such as the ribosomal protein family, and elongation factor 1 and 2 (Table S5).

## Conclusions

This study is the first to investigate the hepatopancreatic function of a swimming crab at the molecular level by 454 pyrosequencing. Our comparative transcriptome produced 53,519 unigenes (including 21,635 contigs and 31,844 singlets) for the swimming crab. Of these unigenes with ORFs, 11,039 sequences were matched to the known species while only 2,773 annotated sequences were identified as belonging to crustacean species. Further analysis revealed 33,427 unigenes with ORFs were found in the hepatopancreas while 25,210 sequences were hepatopancreatic-specific. There were 14, 534, and 22 identified genes involved in food digestion, nutrition metabolism and ovarian development, respectively. Digital expression analysis revealed that 212 differentially expressed genes (DEGs) were found between the growth and endogenous stage hepatopancreas, while 382 DEGs were identified between the endogenous and exogenous stage hepatopancreas. Our results will not only provide valuable genomic information for the understanding of hepatopancreatic roles in nutrition metabolism and ovarian development for *P. trituberculatus*, but also facilitate further investigations of functional genomics for this species and other closely related species.

## Supporting Information

Figure S1 **Crustacean species distribution of the homology search against the nr database.** The number of homologous sequences to each species is shown on the top of each column. (tif)
(TIF)

Figure S2 Distribution of unigene numbers for the major KEGG pathway categories in the *P. trituberculatus* hepatopancreas. The number of unigenes to each category is shown on the top of each column. (tif)
(TIF)

Table S1 Annotation of unigenes in the *P. trituberculatus* hepatopancreas transcriptome. A list of unigenes with the predicted protein sequences, including contigs and singlets, were annotated with biological functions in the hepatopancreas. (xlsx)
(XLSX)

Table S2 Summary of stage-specific KEGG pathways and related unigenes in Hg and Hen & Hex of the *P. trituberculatus* hepatopancreas. Hg: hepatopancreas at growth stage, Hen: hepatopancreas at endogenous vitellogenic stage, Hex: hepatopancreas at exogenous vitellogenic stage. Because Hen and Hex are continuous ovary development process, they were regarded as the whole to compare with Hg. (xlsx)
(XLSX)

Table S3 Summary of candidate genes involved in nutrition metabolism, ovarian development, immunity and other biological processes of the *P. trituberculatus* hepatopancreas. A list of unigenes with the predicted protein sequences, were annotated with functions of nutrition metabolism, ovarian development, immunity and other biological processes in the *P. trituberculatus* hepatopancreas. Hg: hepatopancreas at growth stage, Hen: hepatopancreas at endogenous vitellogenic stage, Hex: hepatopancreas at exogenous vitellogenic stage. (xlsx)
(XLSX)

Table S4 The number of KEGG pathway distribution of DEGs among the different hepatopancreatic transcriptomes of *P. trituberculatus*. The comparisons were conducted between Hen and Hg, and Hex and Hen, including up-regulated pathways and down-regulated pathways. Hg: hepatopancreas at growth stage, Hen: hepatopancreas at endogenous vitellogenic stage, Hex: hepatopancreas at exogenous vitellogenic stage. (pdf)
(PDF)

Table S5 Annotation of major DEGs among the different hepatopancreatic transcriptomes of *P. trituberculatus*. A list of DEGs with the predicted protein sequences, were annotated with biological functions in the hepatopancreas. The comparisons were conducted between Hen and Hg, and Hex and Hen, including up-regulated and down-regulated DEGs. (xlsx)
(XLSX)
