# Supplementary figures and images for: Insights into Hepatopancreatic Functions for Nutrition Metabolism and Ovarian Development in the Crab Portunus trituberculatus: Gene Discovery in the Comparative Transcriptome of Different Hepatopancreas Stages
Source: PLoS One. 2014 Jan 13;9(1):e84921. doi: 10.1371/journal.pone.0084921 (PMC3890295; doi:10.1371/journal.pone.0084921)

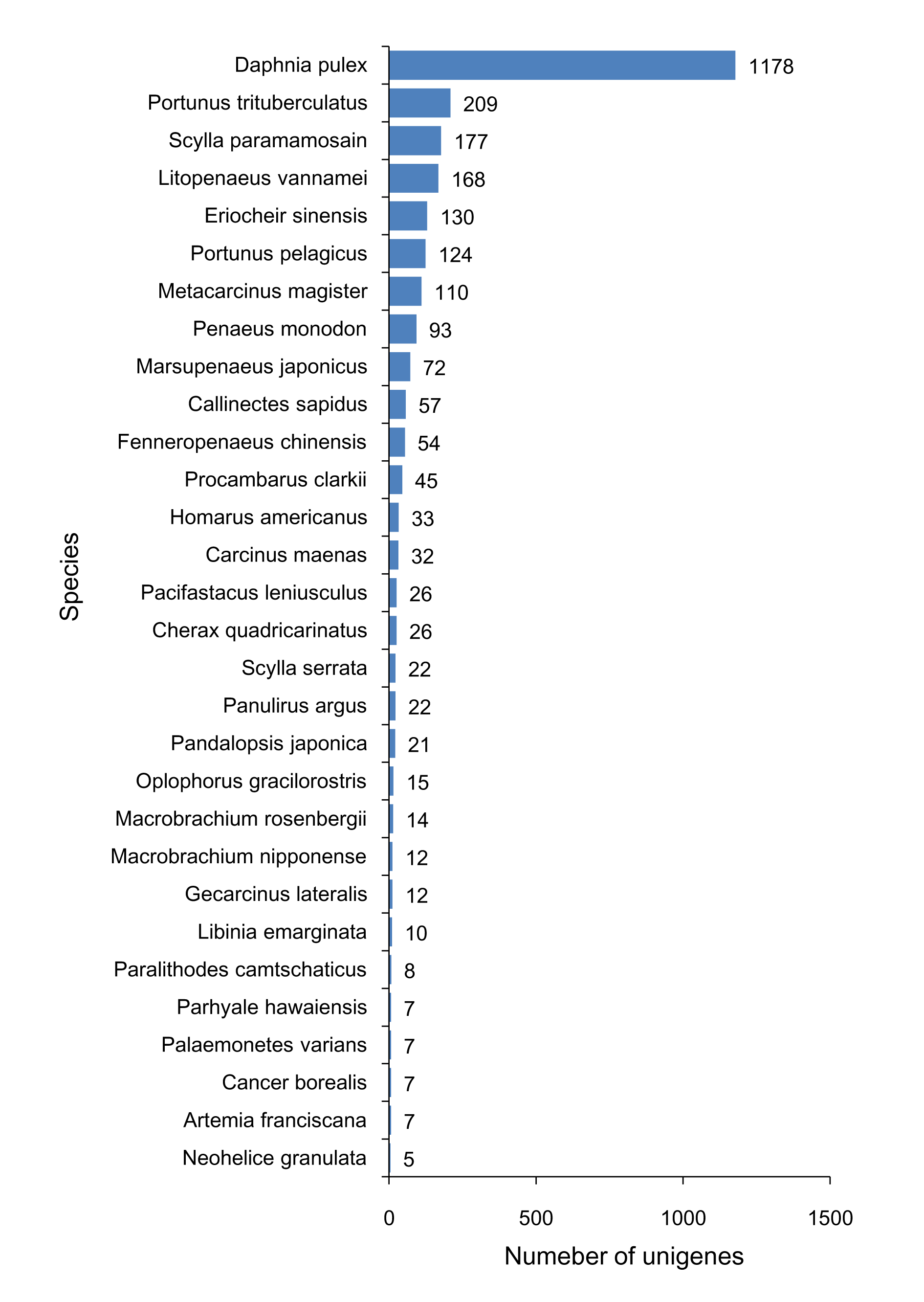

Supplement: Figure S1 — Crustacean species distribution of the homology search against the nr database. The number of homologous sequences to each species is shown on the top of each column. (tif) (TIF) [file pone.0084921.s001.tif]

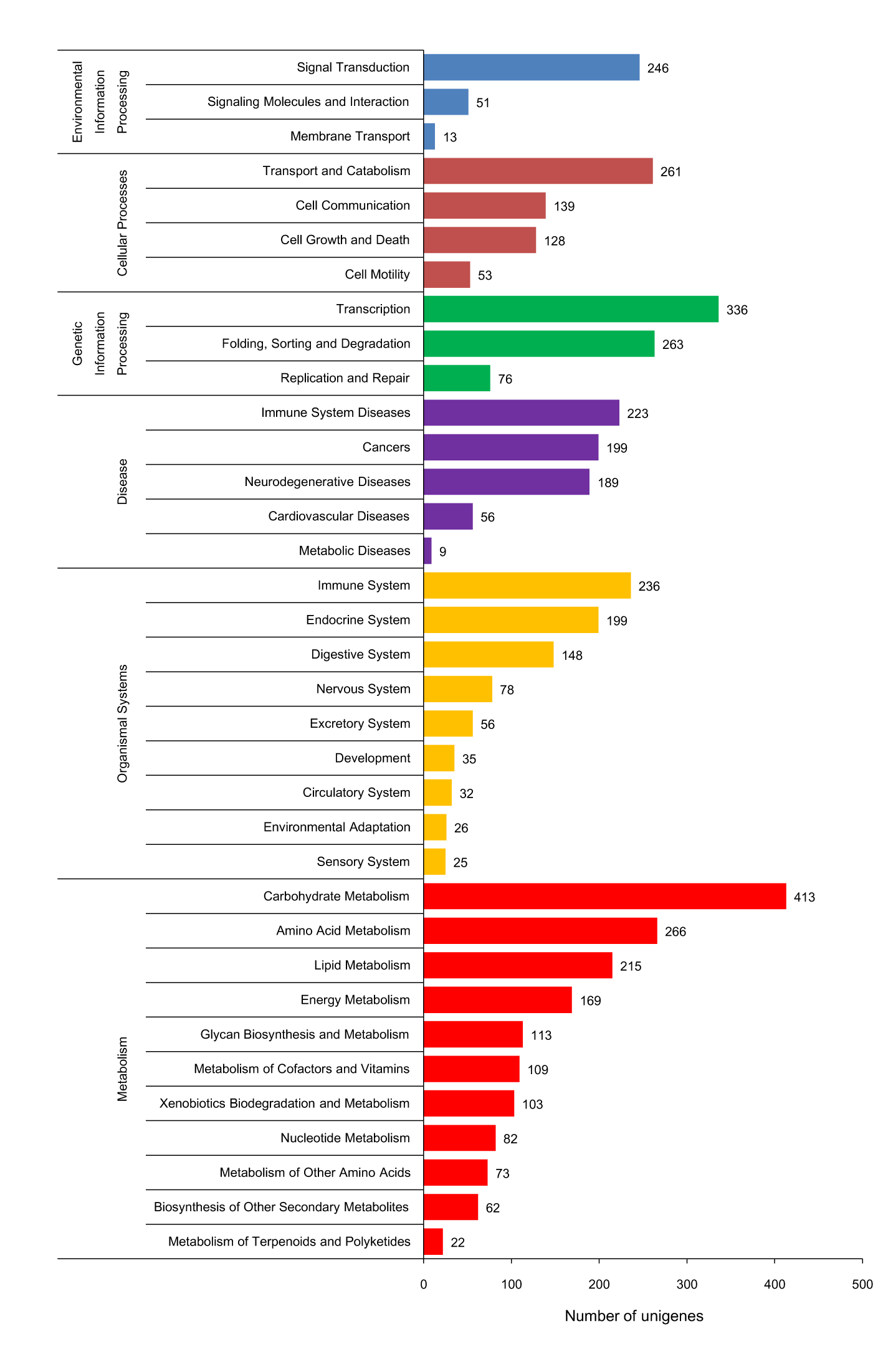

Supplement: Figure S2 — Distribution of unigene numbers for the major KEGG pathway categories in the P. trituberculatus hepatopancreas. The number of unigenes to each category is shown on the top of each column. (tif) (TIF) [file pone.0084921.s002.tif]
