# Supplementary material for: Insights into Hepatopancreatic Functions for Nutrition Metabolism and Ovarian Development in the Crab Portunus trituberculatus: Gene Discovery in the Comparative Transcriptome of Different Hepatopancreas Stages
Source: PLoS One. 2014 Jan 13;9(1):e84921. doi: 10.1371/journal.pone.0084921 (PMC3890295; doi:10.1371/journal.pone.0084921)
Supplement: Table S4 — The number of KEGG pathway distribution of DEGs among the different hepatopancreatic transcriptomes of P. trituberculatus. The comparisons were conducted between Hen and Hg, and Hex and Hen, including up-regulated pathways and down-regulated pathways. Hg: hepatopancreas at growth stage, Hen: hepatopancreas at endogenous vitellogenic stage, Hex: hepatopancreas at exogenous vitellogenic stage. (pdf) (PDF) [file pone.0084921.s006.pdf]

Table S4. The number of KEGG pathway distribution of DEGs among the different hepatopancreatic transcriptomes of *P. trituberculatus*

Pathway class	Pathway	Total number of DEGs in each pathway	Number of DEGs in each pathway in each compare group			
			Hen v Hg* up	Hen v Hg** down	Hex v Hen up	Hex v Hen down
Amino acid metabolism	Alanine, aspartate and glutamate metabolism	2	1	0	0	1
	Arginine and proline metabolism	4	1	1	0	2
	Cysteine and methionine metabolism	5	2	0	0	3
	Glycine, serine and threonine metabolism	9	3	1	1	4
	Tyrosine metabolism	10	2	2	3	3
	Valine, leucine and isoleucine degradation	2	0	1	1	0
Biosynthesis of other secondary metabolites	Betalain biosynthesis	10	2	2	3	3
	Isoquinoline alkaloid biosynthesis	10	2	2	3	3
Cancers	Acute myeloid leukemia	1	0	1	0	0
	Chronic myeloid leukemia	1	0	1	0	0
	Colorectal cancer	1	0	1	0	0
	Endometrial cancer	1	0	1	0	0
	Glioma	1	0	1	0	0
	Melanoma	1	0	1	0	0
	Non-small cell lung cancer	1	0	1	0	0
	Pancreatic cancer	1	0	1	0	0
	Pathways in cancer	1	0	1	0	0
	Prostate cancer	1	0	1	0	0
	Renal cell carcinoma	1	0	1	0	0
	Small cell lung cancer	1	0	1	0	0
Carbohydrate metabolism	Amino sugar and nucleotide sugar metabolism	8	4	0	0	4
	Ascorbate and aldarate metabolism	1	0	0	1	0
	Citrate cycle (TCA cycle)	1	0	0	0	1
	Fructose and mannose metabolism	4	1	1	1	1
	Glycolysis / Gluconeogenesis	3	0	1	2	0
	Glyoxylate and dicarboxylate metabolism	1	0	0	0	1
	Inositol phosphate metabolism	1	0	0	1	0
	Pentose and glucuronate interconversions	1	0	0	1	0
	Pentose phosphate pathway	2	0	1	1	0
	Propanoate metabolism	1	0	0	1	0
	Pyruvate metabolism	1	0	0	1	0
	Starch and sucrose metabolism	2	0	1	1	0

Pathway class	Pathway	Total number of DEGs in each pathway	Number of DEGs in each pathway in each compare group			
			Hen v Hg* up	Hen v Hg** down	Hex v Hen up	Hex v Hen down
Cardiovascular diseases	Arrhythmogenic right ventricular cardiomyopathy (ARVC)	1	0	0	0	1
	Dilated cardiomyopathy (DCM)	1	0	0	0	1
	Hypertrophic cardiomyopathy (HCM)	1	0	0	0	1
	Viral myocarditis	1	0	0	0	1
Cell communication	Adherens junction	1	0	0	0	1
	Focal adhesion	4	1	1	1	1
	Gap junction	1	0	0	1	0
	Tight junction	1	0	0	0	1
Cell growth and death	Apoptosis	1	0	1	0	0
	Meiosis - yeast	1	1	0	0	0
	Oocyte meiosis	1	1	0	0	0
Cell motility	Regulation of actin cytoskeleton	6	2	1	1	2
Circulatory system	Cardiac muscle contraction	1	0	0	1	0
	Vascular smooth muscle contraction	1	1	0	0	0
Development	Axon guidance	1	0	0	0	1
Digestive system	Carbohydrate digestion and absorption	4	1	2	0	1
	Pancreatic secretion	6	3	0	0	3
	Protein digestion and absorption	5	2	0	0	3
Endocrine system	Insulin signaling pathway	6	2	2	0	2
	Melanogenesis	10	2	2	3	3
	PPAR signaling pathway	1	0	0	1	0
	Progesterone-mediated oocyte maturation	1	0	1	0	0
Energy metabolism	Carbon fixation in photosynthetic organisms	3	0	1	2	0
	Methane metabolism	3	0	2	1	0
	Nitrogen metabolism	4	2	0	0	2
	Oxidative phosphorylation	3	0	0	2	1
	Reductive carboxylate cycle (CO2 fixation)	1	0	0	0	1
Environmental adaptation	Plant-pathogen interaction	1	0	1	0	0
Excretory system	Aldosterone-regulated sodium reabsorption	1	0	1	0	0
	Collecting duct acid secretion	1	0	0	0	1
Folding, sorting and degradation	Protein processing in endoplasmic reticulum	4	0	1	3	0

Pathway class	Pathway	Total number of DEGs in each pathway	Number of DEGs in each pathway in each compare group			
			Hen v Hg* up	Hen v Hg** down	Hex v Hen up	Hex v Hen down
Glycan biosynthesis and metabolism	Glycosaminoglycan degradation	2	1	0	0	1
	Glycosphingolipid biosynthesis - ganglio series	2	1	0	0	1
	Glycosphingolipid biosynthesis - globo series	2	1	0	0	1
	O-Glycan biosynthesis	1	0	0	0	1
	Other glycan degradation	2	0	0	0	1
Immune system	Antigen processing and presentation	5	1	1	2	1
	B cell receptor signaling pathway	1	0	1	0	0
	Chemokine signaling pathway	2	1	1	0	0
	Fc epsilon RI signaling pathway	1	0	1	0	0
	Fc gamma R-mediated phagocytosis	4	0	2	1	1
	Leukocyte transendothelial migration	2	0	1	0	1
	Natural killer cell mediated cytotoxicity	1	0	1	0	0
	T cell receptor signaling pathway	1	0	1	0	0
	Toll-like receptor signaling pathway	1	0	1	0	0
Infectious diseases	Amoebiasis	1	0	1	0	0
	Bacterial invasion of epithelial cells	3	0	1	1	1
	Chagas disease	2	0	1	1	0
	Epithelial cell signaling in Helicobacter pylori infection	1	0	0	0	1
	Hepatitis C	1	0	1	0	0
	Pathogenic Escherichia coli infection	3	0	0	2	1
	Shigellosis	2	0	0	1	1
	Toxoplasmosis	1	0	1	0	0
	Vibrio cholerae infection	2	0	0	0	2
Lipid metabolism	Arachidonic acid metabolism	1	0	1	0	0
	Biosynthesis of unsaturated fatty acids	1	0	0	1	0
	Glycerophospholipid metabolism	2	0	0	2	0
	Sphingolipid metabolism	1	1	0	0	0
	Steroid biosynthesis	4	0	2	1	1
	Steroid hormone biosynthesis	1	0	0	1	0
Metabolic diseases	Type II diabetes mellitus	2	0	1	1	0
Metabolism of cofactors and vitamins	Folate biosynthesis	1	0	0	0	1
	One carbon pool by folate	2	1	0	0	1
	Porphyrin and chlorophyll metabolism	2	0	0	1	1

Pathway class	Pathway	Total number of DEGs in each pathway	Number of DEGs in each pathway in each compare group			
			Hen v Hg* up	Hen v Hg** down	Hex v Hen up	Hex v Hen down
Metabolism of cofactors and vitamins	Retinol metabolism	1	0	0	1	0
	Riboflavin metabolism	10	2	2	3	3
	Glycerolipid metabolism	2	1	0	0	1
Metabolism of other amino acids	Selenoamino acid metabolism	3	1	0	0	2
Nervous system	Long-term potentiation	1	1	0	0	0
	Neurotrophin signaling pathway	1	0	1	0	0
Neurodegenerative diseases	Alzheimer's disease	2	0	0	2	0
	Huntington's disease	3	0	0	2	1
	Parkinson's disease	3	0	0	2	1
Nucleotide metabolism	Purine metabolism	3	0	0	2	1
	Pyrimidine metabolism	1	0	0	0	1
Replication and repair	Nucleotide excision repair	1	0	1	0	0
Sensory system	Phototransduction - fly	1	0	0	0	1
Signal transduction	Calcium signaling pathway	1	0	0	0	1
	ErbB signaling pathway	1	0	1	0	0
	Jak-STAT signaling pathway	1	0	1	0	0
	MAPK signaling pathway	3	2	0	0	1
	mTOR signaling pathway	2	0	1	0	1
	Phosphatidylinositol signaling system	1	0	1	0	0
	Two-component system	3	1	0	0	2
	VEGF signaling pathway	1	0	1	0	0
Signaling molecules and interaction	Neuroactive ligand-receptor interaction	3	1	0	0	2
Translation	Ribosome	25	5	1	0	19
	RNA transport	2	1	0	0	1
Transport and catabolism	Endocytosis	1	0	1	0	0
	Lysosome	9	1	2	1	5
	Phagosome	6	0	1	3	2
Xenobiotics biodegradation and metabolism	Aminobenzoate degradation	1	0	0	0	1
	Drug metabolism - cytochrome P450	1	0	0	1	0
	Drug metabolism - other enzymes	1	0	0	1	0
	Metabolism of xenobiotics by cytochrome P450	1	0	0	1	0

* Hen v Hg up: DEGs are up- regulated in Hen, ** Hen v Hg down: DEGs are down- regulated in Hen
